# Kea (*Nestor notabilis*) fail a loose-string connectivity task

**DOI:** 10.1038/s41598-021-94879-x

**Published:** 2021-07-29

**Authors:** Amalia P. M. Bastos, Patrick M. Wood, Alex H. Taylor

**Affiliations:** School of Psychology, The University of Auckland, Private Bag 92019, Auckland, 1142 New Zealand

**Keywords:** Psychology, Animal behaviour

## Abstract

Naïve individuals of some bird species can rapidly solve vertical string-pulling tasks with virtually no errors. This has led to various hypotheses being proposed which suggest that birds mentally simulate the effects of their actions on strings. A competing embodied cognition hypothesis proposes that this behaviour is instead modulated by perceptual-motor feedback loops, where feedback of the reward moving closer acts as an internal motivator for functional behaviours, such as pull-stepping. To date, the kea parrot has produced some of the best performances of any bird species at string-pulling tasks. Here, we tested the predictions of the four leading hypotheses for the cognition underpinning bird string-pulling by presenting kea with a horizontal connectivity task where only one of two loose strings was connected to the reward, both before and after receiving perceptual-motor feedback experience. We find that kea fail the connectivity task both before and after perceptual-motor feedback experience, suggesting not only that kea do not mentally simulate their string-pulling actions, but also that perceptual-motor feedback alone is insufficient in eliciting successful performance in the horizontal connectivity task. This suggests a more complex interplay of cognitive factors underlies this iconic example of animal problem-solving.

## Introduction

String-pulling behaviour occurs naturally in birds and has been reported in a range of species, being especially prevalent across passerines, particularly corvids, and parrots^1^. This behaviour describes subjects pulling on a baited string to bring a reward closer, and holding it down with their foot, preventing the reward from falling back down. The swiftness and efficiency of this behaviour in naïve individuals has been attributed by different researchers as evidence for various cognitive mechanisms including planning, means-end understanding, and insight (an immediate understanding of the problem without prior experience)^1–3^. All three of these hypotheses would predict that, upon seeing a string with a reward at one end, subjects can mentally simulate the effects of their pulling actions on the string, imagining how they might move the reward closer to themselves. Therefore, upon seeing two strings, only one of which is baited, the same hypotheses predict that animals can mentally simulate their pulling actions on both options, and therefore select the correct string above chance, without trial-and-error.

Contrary to these hypotheses, the perceptual-motor feedback loop hypothesis suggests that, upon encountering a baited string for the first time, subjects will perform exploratory behaviours, and when a set of behaviours—such as pull-stepping—brings the reward on the end of the string closer, or keep it in place, they continue to perform these behaviours, such that they become self-reinforcing and increase in both frequency and efficiency over time^4–6^. Therefore, unlike several other cognitive mechanisms suggested for string-pulling, this embodied cognition hypothesis does not require mental modelling or simulating the effects of subjects’ actions on a string^4^. Rather, it relies upon subjects having sufficiently large associative brain areas or more connected perceptual and motor pathways to detect and link the effects of their actions while coordinating pull-step actions, or both^4^.

The perceptual-motor feedback loop account therefore predicts that subjects without string-pulling experience should fail at tasks where pulling on the string provides no feedback, that is, does not cause the food reward to move closer to the subject^4,5^. To date, only naïve New Caledonian crows (*Corvus moneduloides*) have been presented with tasks that directly test this claim, as studies with other species have given subjects experience of this feedback loop before test^7–18^. In one study, naïve New Caledonian crows struggled to pull up a string when the end of the string was visually restricted, which prevented them from perceiving the distance between themselves and the reward^4^. In contrast, crows that had previously experienced the feedback loop in a typical string-pulling task learned to solve this problem.

Two studies that have challenged the perceptual-motor feedback hypothesis used the pull-down test with ravens^3^ and green-winged macaws^19^. In both cases, groups of individuals that were either naïve to or experienced with vertical string-pulling were presented with a string-pulling task that required a counter-intuitive motor action: subjects had to pull down on a string looping over a higher perch before coming back down. Inexperienced ravens and both groups of macaws failed at this task, despite the food at the end of the string moving up when they performed a downward pull. Although both studies interpret this as evidence that a perceptual-motor feedback loop is not sufficient for acquiring string-pulling behaviour^3,19^, it is unclear if subjects experienced it as such: when reaching up with their bills to pull down on the string, their eyes would have been directed upwards, and so it is possible that subjects never observed the reward moving closer. Therefore, these studies could also be interpreted as providing evidence for the perceptual-motor feedback hypothesis.

Finally, support for the feedback loop hypothesis has also emerged from a connectivity task with naïve New Caledonian crows. When crows with no experience of vertical string-pulling chose between a meandering horizontal continuous string and a similarly-positioned broken string, both of which contained food at their ends, they showed no preference for either string^5^. This horizontal loose-string connectivity task, or broken-string task, is particularly interesting because in all cases where avian subjects have succeeded at this task, they previously experienced a perceptual-motor feedback loop with vertical strings^8–14^. This pattern also appears to extend beyond birds to other string-pulling tasks devoid of feedback: for example, bumblebees with feedback-loop experience successfully pull on coiled strings, but naïve bees and bees that have only observed string-pulling by others (and therefore did not experience feedback themselves), do not^20^.

A key question that remains unanswered is whether naïve New Caledonian crows’ failure at the loose-string connectivity task is directly attributable to their lack of perceptual-motor feedback loop experience. Given the pattern of results present in the literature, we raise a feedback experience hypothesis: that birds must first experience perceptual-motor feedback with strings to learn to attend to the ends of the strings, which in turn could allow them to succeed at two-string discrimination tasks such as those testing connectivity, contact, and continuity.

Kea (*Nestor notabilis*) are an ideal model species for testing this feedback experience hypothesis. Kea spontaneously solve the single vertical string task on their first trial and succeed in the crossed-strings task, where the end of the baited string is located under the starting point of the unbaited one^7,21^. In contrast, experienced New Caledonian crows perform at chance at this test of causal understanding^4^, suggesting kea might outperform them at string-pulling tasks generally. Furthermore, a previous study showed that two of five kea selected a connected over an unconnected board within their first trial, and one of them selected the correct option in all of their first 10 trials^22^. Although the other subjects took longer to understand the nature of this task, this result suggests that kea are capable of understanding connectivity without extensive training, at least in contexts unrelated to string-pulling.

We therefore tested naïve kea (*Nestor notabilis*) on three experiments to test both the predictions of the three mental simulation hypotheses, and the feedback experience hypothesis we propose. In Experiment 1, kea were presented with the horizontal connectivity discrimination task previously presented to New Caledonian crows, to establish whether kea without any perceptual-motor feedback loop experience in string-pulling contexts were capable of understanding the nature of this task. In Experiment 2, kea experienced 10 vertical string-pulling trials, replicating a previous study with another captive kea population. Then, following an additional 10 trials of vertical string-pulling experience, in Experiment 3 kea were presented with a repeat of the first experiment. If experience of perceptual-motor feedback explains the pattern of results in the literature up to this point, enabling birds to attend to the ends of strings, then kea should fail at Experiment 1 but succeed at Experiment 3, following experience of vertical string-pulling.

## Results

None of our subjects performed above chance across the 20 trials of Experiment 1 (Table 1). Overall, kea attempted to change their original choice in 41 of 140 trials (29.3% of the time), and of these switches, only 9 were attempted in the wrong direction (attempting to switch to the incorrect choice after selecting the correct choice; 22.0%). Unlike New Caledonian crows^5^, kea persisted in their interactions with the apparatus and strings, even when they did not completely retrieve them from the apparatus. Strings were fully retrieved by subjects in 112 of 140 trials (80.0%) and across all trials subjects spent an average of 18.54 ± 19.70 s interacting with the strings. When strings were not fully retrieved, there was no consistent pattern across individuals as to whether this was the correct or incorrect string (Table 1). Unlike New Caledonian crows, all subjects continued to interact with strings even in failed trials (averaging 22.36 ± 21.35 s in incorrect trials) and completed the experiment with no refusals.

**Table 1:** Subjects’ performance in Experiment 1, with columns showing, in order: number of correct choices (measured as first touch) for the continuous string (Bayesian binomial test values in parentheses), number of times subjects first made a correct choice and then switched to the incorrect choice (counted as correct and ended the trial), number of times subjects made the incorrect choice and then switched to the correct choice (counted as incorrect and ended the trial), number of times subjects did not fully retrieve the chosen string from the apparatus and how many of these unretrieved strings were the correct choice, and average time spent interacting with strings.

Subject	Correct choices	Correct switches	Incorrect switches	Unretrieved strings	Interaction time
Blofeld	10/20 (BF = 0.270)	1	4	10 (6 correct)	32.68 ± 23.93 s
Bruce	11/20 (BF = 0.297)	4	6	4 (4 correct)	11.74 ± 12.76 s
Loki	12/20 (BF = 0.396)	0	4	0	9.01 ± 16.77 s
Moriarty	9/20 (BF = 0.297)	0	3	2 (0 correct)	24.81 ± 22.97 s
Neo	7/20 (BF = 0.644)	2	7	4 (2 correct)	18.68 ± 18.02 s
Plankton	7/20 (BF = 0.644)	1	5	7 (2 correct)	18.66 ± 19.33 s
Taz	11/20 (BF = 0.297)	1	3	1 (1 correct)	14.19 ± 13.49 s

In Experiment 2, our subjects performed similarly to the kea population used in a previous study^7^. All subjects succeeded in retrieving the rewarding token hanging on the end of the vertical string, with the exception of Bruce, whose missing upper bill made manipulating the vertical string too challenging (Table 2). As in previous research^7^, most kea rapidly solved the task from their first or second trial, with the average (mean) duration of individuals’ first successful trial being higher than that for subsequent successful trials (Table 3). Solution times were similar to those in the previous study (83.10 ± 128.39 s in previous study; 71.36 ± 42.88 s in present study; Bayesian two-tailed independent samples t-test, BF_10_ = 0.705).

**Table 2:** Individuals’ performance in Experiment 2, namely: the number of successful retrievals performed, the average time taken to retrieve the token across all of their successful trials, and each individual’s pull-step ratio across all their successful trials.

	Successful retrievals	Mean trial duration	Pull-step ratio
Blofeld	6	25.14 ± 31.12 s	89.17 ± 12.01%
Bruce	0	n/a	n/a
Loki	9	9.31 ± 2.78 s	91.76 ± 10.29%
Megatron	10	20.76 ± 29.09 s	82.00 ± 14.14%
Moriarty	10	12.25 ± 13.57 s	86.67 ± 14.27%
Neo	10	19.56 ± 41.98 s	95.50 ± 9.56%
Plankton	10	16.49 ± 14.12 s	85.81 ± 12.97%
Taz	10	15.38 ± 26.16 s	95.50 ± 9.56%

**Table 3:** The first column shows the average duration across all individuals’ successful trials in Experiment 2, measured as the time taken from touching the string for the first time to holding the black token.

Trial number	Mean duration of successful trials	Pull-step ratio of successful trials
1	71.36 ± 42.88 s	83.10 ± 12.49%
2	10.66 ± 3.82 s	88.33 ± 11.18%
3	8.37 ± 2.67 s	93.57 ± 11.07%
4	9.99 ± 4.57 s	83.10 ± 17.01%
5	10.45 ± 3.70 s	84.76 ± 10.82%
6	10.30 ± 4.75 s	94.13 ± 11.04%
7	6.95 ± 3.11 s	95.83 ± 10.21%
8	7.08 ± 1.22 s	100.00 ± 0.00%
9	10.13 ± 4.78 s	83.61 ± 13.60%
10	7.35 ± 1.16 s	90.00 ± 13.69%

Unsuccessful trials where individuals failed to retrieve the rewarding token are not included. The second column contains the pull-step ratios across individuals their successful trials, calculated as the percentage of correct pulls followed by steps (or other string attachments) over all attempts to pull the vertical string.

On average across all trials, subjects performed 3.35 ± 0.93 correct pull-steps, and only 0.49 ± 0.59 errors per trial. Kea therefore exhibited high pull-step ratios in their successful trials (mean across all subjects in all successful trials: 89.47 ± 12.44%), which was similar to the equivalent measure in New Caledonian crows (90.2 ± 2.42%) ^4^. Pull-step ratios were high from the first successful trial (mean across all subjects’ first trials: 83.10 ± 12.49%). The number of pull-steps performed in their first successful trial did not differ significantly from the number of pull-steps in their last successful trial (mean for first trial across all subjects: 3.57 ± 0.98 pull-steps; mean for final trial: 2.80 ± 0.45 pull-steps; Bayesian two-tailed paired samples t-test, BF_10_ = 0.522). We found no evidence to suggest that the group’s performance improved with experience across all test trials (Bayesian correlation, n = 7, Pearson’s r = − 0.553, BF_10_ = 1.308), despite the reduced solution time between the first two successful trials, mirroring the results from previous work on another kea population^7^. Therefore, trial duration averages indicate that kea rapidly achieved ceiling performance from their second vertical string-pulling trial onwards, rather than making gradual improvements to their performance over several trials.

Experiment 3 was a direct replication of Experiment 1 with the five individuals that experienced both the loose-string connectivity task of Experiment 1 and successfully retrieved the vertical string in at least one trial of Experiment 2. This did not include one subject, Bruce, that was unable to pull up the string due to his missing upper bill. None of the five subjects in this study performed above chance following experience of the perceptual-motor feedback loop (Table 4). Subjects attempted to switch their choices in 28 out of 120 trials (23.3%), with only one of these switches being from a correct to an incorrect choice (3.57% of switches, compared to 21.95% in Experiment 1). As in Experiment 1, subjects continued to interact with the strings even when they made incorrect choices, averaging 20.20 ± 12.91 s across all trials.

**Table 4:** Subjects’ performance in Experiment 3, with columns showing, in order: number of correct choices for the continuous string (Bayesian binomial test values in parentheses), number of times subjects first made a correct choice and then switched to the incorrect choice (counted as correct and ended the trial), number of times subjects made the incorrect choice and then switched to the correct choice (counted as incorrect and ended the trial), number of times subjects did not fully retrieve the chosen string from the apparatus, and average time spent interacting with strings.

Subject	Correct choices	Correct switches	Incorrect switches	Unretrieved strings	Interaction time
Blofeld	10/20 (BF = 0.270)	1	1	0	33.89 ± 22.61 s
Loki	11/20 (BF = 0.297)	0	8	0	5.26 ± 12.91 s
Moriarty	9/20 (BF = 0.297)	0	2	0	26.21 ± 23.22 s
Neo	10/20 (BF = 0.270)	0	7	0	20.09 ± 19.90 s
Plankton	13/20 (BF = 0.644)	0	3	1 (1 correct)	23.47 ± 22.98 s
Taz	11/20 (BF = 0.297)	0	6	0	12.29 ± 16.64 s

## Discussion

Our study tested whether experience of a perceptual-motor feedback loop during string-pulling might explain the pattern of results observable in the bird literature: naïve subjects fail at loose-string connectivity tasks^5^, and experienced birds sometimes succeed at it^8–14^. Kea failed this task both before and after receiving experience of vertical string-pulling, that is, regardless of their experience with perceptual-motor feedback loops. This result both opposes the predictions of the insight, planning, and means-end understanding hypotheses which predict an immediate understanding of string-pulling problems without perceptual-motor feedback experience, but also shows that feedback experience alone cannot elicit success at the horizontal loose-string connectivity task.

It is unlikely that the failure of kea at both connectivity experiments reflects a general discrepancy in cognitive or motor abilities in our population of kea. In fact, the results of Experiment 2 reveal that our population of kea behaved very similarly to conspecifics in another captive population^7,21^ and wild New Caledonian crows^4,5^. As in the original kea study^7^, our subjects quickly learned to pull up vertical strings, showing few errors and ceiling performances after their first successful trial.

Our results also demonstrate that kea’s failure was not a result of hesitancy to interact with the strings. Unlike crows^5^, kea persisted in their interactions with the strings throughout Experiments 1 and 3, and we never observed choice refusals in any trials. As a highly neophilic species^23^, it is possible that, even after having failed to obtain the reward at the end of the string, kea were still interested in the properties of this novel material and therefore continued to interact with it. This is consistent with our observation that subjects often chewed on the ends of the strings even following incorrect choices, suggesting not all their interactions with the strings were made with the intent to retrieve the out-of-reach black token. This issue was not observed in Experiment 2 presumably because either goal (obtaining the black token or playing with the string) could have equally resulted in accurate and efficient step-pulling behaviour by the subjects. Kea became more persistent in Experiment 3 than they were in Experiment 1, leaving fewer strings unretrieved in the final experiment. This may have been a consequence of their increased experience performing multiple pulling actions on the vertical string presented in Experiment 2. However, their vertical string experience did not improve their ability to discriminate between the connected and unconnected strings in their second attempt at the horizontal connectivity task. Their inability to make this discrimination is unlikely to be a consequence of kea finding it difficult to make choices between the two options presented. This same population of kea have previously demonstrated an ability to make binary choices between highly similar stimuli^24–26^, and subjects were specifically trained to make choices using the apparatus of these experiments prior to test.

Our results demonstrate that although kea made fewer switches from correct to incorrect choices in their second attempt at the loose-string connectivity task, they still initially selected the correct string at chance. This would suggest that although kea realised that they made an incorrect choice after attempting to pull on the unconnected string, they did not simulate their actions on horizontal strings prior to their first touch, even after ample experience with the setup. Therefore, kea’s persistent failure at the connectivity task provides evidence against the insight, planning, and means-understanding hypotheses for string-pulling behaviour, all of which would predict an ability to mentally simulate the consequences of their actions on strings both with and without experience of perceptual-motor feedback loops. This finding is notable in light of research suggesting that kea are capable of mentally representing objects in other contexts^25,26^.

Despite the pattern of results in the literature showing that individuals of several bird species succeed at string connectivity tasks following perceptual-motor feedback with vertical strings^8–14^, while naïve individuals fail^5^, we did not find evidence that experience of feedback improves performance on a string connectivity task in kea. This is particularly puzzling given that previous studies have shown that kea can successfully distinguish connected from unconnected wooden boards^22^. As such, further work is required to establish whether birds’ understanding of connectivity is context-dependent, by comparing performance before and after feedback experience both during string-pulling and in tasks using materials other than string. This could shed light on the interplay of contextual variables and cognitive mechanisms that underpin performance on this iconic problem-solving task.

## Methods

### Subjects

Our subjects were eight captive kea housed at Willowbank Wildlife Reserve (Table 5). In both experiments, strings were attached to black tokens, which kea had been previously trained to exchange for a food reward^25,26^. None of the subjects had any prior experience with strings. Research was carried out with approval from the University of Auckland ethics committee (reference number 001816) and all methods were carried out in accordance with the relevant guidelines and regulations. The study was also carried out in compliance with ARRIVE guidelines.

**Table 5:** Individuals’ hatch dates, sex, and participation in the two experiments. All subjects were parent reared.

Subject	Hatch date (known or estimated)	Sex	Participation
Blofeld	August 2013	M	Experiments 1–3
Bruce	January 2012	M	Experiments 1 & 2
Loki	August 2014	M	Experiments 1–3
Megatron	October 2019	M	Experiment 2
Moriarty	August 2014	M	Experiments 1–3
Neo	September 2012	M	Experiments 1–3
Plankton	August 2014	M	Experiments 1–3
Taz	September 2012	M	Experiments 1–3

### Experiment 1

**Figure 1:**
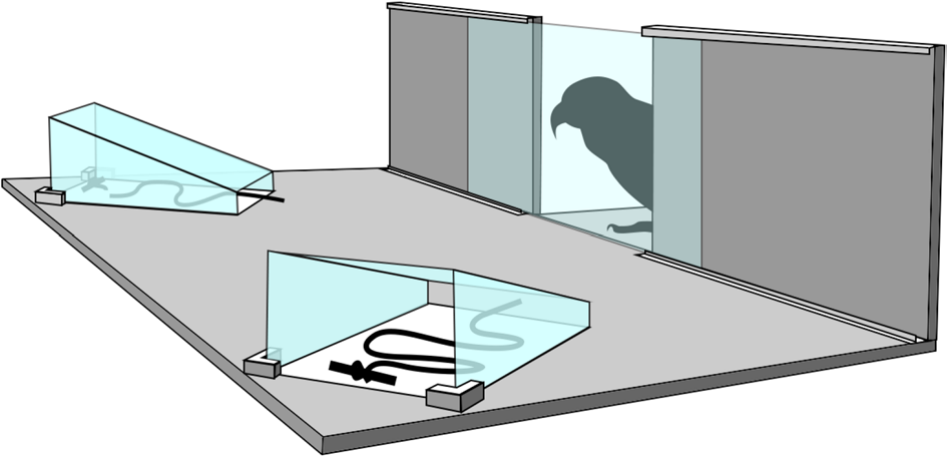
Diagram of setup used in Experiment 1, where kea had to choose between a continuous and a broken string, both of which were placed under sloping acrylic shields and were attached to rewarding black tokens.

Trials were conducted on a rectangular platform measuring 65 cm × 130 cm, with a centrally protruding 30 cm × 30 cm shelf where subjects could stand behind an acrylic sheet between trials, serving as subjects’ starting position in all trials (Fig. 1). Two 20 cm × 30 cm curved acrylic shields were placed diagonally on the platform, equidistant to the starting position. Kea were first habituated to the apparatus and trained to make choices between options presented under the two shields, neither of which contained strings or required pulling. At test, two horizontal string-pulling options were presented under the two shields, with only their tips accessible at the front. As in the study with New Caledonian crows^5^, kea had to choose between a continuous piece of string attached to a rewarding black token at the far end, or a broken string missing a 10 cm section which was otherwise identically arranged (Fig. 1). The side on which the continuous (correct) option was presented was pseudorandomised and counterbalanced, with no more than two trials in a row where the continuous string was placed on the same side, for a total of 20 trials, presented in blocks of 10 trials. The experimenter wore mirrored sunglasses and was kept blind to experimental hypotheses across all trials, so as to not unintentionally cue subjects at test.

Each trial began with the subject standing at the starting position, behind the closed plexiglass barrier, for an observation period of 20 s, during which subjects could see but not approach the two options. After 20 s, the experimenter opened the plexiglass barrier and stepped back, allowing subjects to step towards either plexiglass shield. Subjects were allowed to make only one choice, and the trial ended when the kea either: (a) obtained the black token from the continuous string, which they were then allowed to exchange for a food reward, (b) interacted with one string and then attempted to interact with another without obtaining either token, (c) interacted with either string without obtaining the continuous string’s token for 1 min, or (d) refused to touch or interact with either choice for 3 min.

### Experiment 2

To ensure that our population of kea performed typically in a vertical string-pulling task, subjects experienced ten trials of a single continuous vertical cotton string. The platform for this experiment consisted of a 30 cm × 30 cm base with a ⌀40 mm perch which overhung from one side by 41 cm. A 70 cm long piece of string (the same length as used in a previous study^7^) with a black token attached at one end hung 20 cm from the edge. Each trial was 3 min long, during which time the subject was allowed to interact with the perch and the string. Failure to pull the token up within the duration of a trial was counted as a failure and leaving the platform mid-trial was counted as a refusal. If subjects failed or refused a trial, that block was interrupted and resumed at a later time. Where subjects succeeded at a trial, they were given the next trial immediately, for a total of up to ten trials.

### Experiment 3

Experiment 3 was an exact replication of Experiment 1, excluding the only individual (Bruce) that did not experience the perceptual-motor feedback loop in Experiment 2 due to being physically unable to pull up the string.

### Video coding and analyses

Trials were filmed and coded in terms of subjects’ binary choices (Experiments 1 and 3) or the duration of successful trials (Experiment 2). Successful trials in Experiment 2 (where the subject retrieved the black token from the vertical string) were also coded for number of pulls, steps, and errors. Errors occurred when subjects: (a) failed to step on the string following a pull, (b) failed to otherwise secure the string following a pull, (c) mis-coordinated a step after a pull, which failed to secure the string, and (d) stopped pull-step actions and released the string before the token was successfully retrieved. Their behaviours were used to calculate a pull-step ratio^4^, which consisted of the number of correct pull-steps over the total number of pulls attempted by the subject. Any manipulation of the string prior to the first pull were ignored^4^, as these usually consisted of exploratory behaviours such as touching or biting the string where it was attached to the perch. Unlike New Caledonian crows, kea used other forms of string attachment as an alternative to stepping (such as swinging the string over the side of the perch, which also held it in place). These were counted as steps for the purposes of the pull-step ratio.

Success in Experiment 1 was analysed at the individual level, with Bayesian two-tailed binomial tests with default beta priors set at 0.5. We also correlated successful trial number (counting from the first successful trial onwards) and trial duration for Experiment 2 at the group level, using a Bayesian correlation with default stretched beta prior width of 1. We used two-tailed Bayesian t-tests to compare performances both within Experiment 2 and between our results and those of a previous study^7^. Bayes factors below 0.33 and above 3 were taken as substantial evidence for the null hypothesis or alternative hypothesis, respectively^27^. All analyses were carried out in JASP v.0.13.1.0^28^.

### Ethics statement

This research was conducted under ethics approval from The University of Auckland Ethics Committee (reference number 001816).

## Supplementary Information

Supplementary Dataset.

## Data Availability

All supporting data is available as an electronic supplementary material.
